# Early in-hospital initiation of angiotensin-receptor–neprilysin inhibitor in post-acute myocardial infarction patients with impaired left ventricular systolic function: a systematic review and meta-analysis of randomized controlled trials

**DOI:** 10.3389/fcvm.2026.1877075

**Published:** 2026-07-21

**Authors:** Quynh Nguyen, Trang Thi Quynh Tran, Chia-Te Liao, Chung-Lieh Hung, Hung-Yu Chang, Chih-Wei Chen, Yi-Cheng Lin, Chun-Yao Huang, Chien-Yi Hsu

**Affiliations:** 1 College of Medicine, Taipei Medical University, Taipei, Taiwan; 2 Department of Rehabilitation, Hue University of Medicine and Pharmacy, Hue University, Hue, Vietnam; 3 AI Biomedical Research Institute, Kaohsiung Medical University, Kaohsiung, Taiwan; 4 The One AITech, Taipei, Taiwan; 5 Division of Cardiology, Department of Internal Medicine, MacKay Memorial Hospital, Taipei, Taiwan; 6 Institute of Biomedical Sciences, MacKay Medical University, New Taipei, Taiwan; 7 Faculty of Medicine, College of Medicine, National Yang Ming Chiao Tung University, Taipei, Taiwan; 8 The Industrial Doctorate Program in Smart Healthcare Management and Policy, Institute of Hospital and Health Care Administration, National Yang Ming Chiao Tung University, Taipei, Taiwan; 9 Heart Center, Cheng Hsin General Hospital, Taipei, Taiwan; 10 Division of Cardiology and Cardiovascular Research Center, Department of Internal Medicine, Taipei Medical University Hospital, Taipei, Taiwan; 11 Division of Cardiology, Department of Internal Medicine, School of Medicine, College of Medicine, Taipei Medical University, Taipei, Taiwan; 12 Taipei Heart Institute, Taipei Medical University, Taipei, Taiwan; 13 Department of Pharmacy, Taipei Medical University Hospital, Taipei, Taiwan; 14 School of Pharmacy, College of Pharmacy, Taipei Medical University, Taipei, Taiwan

**Keywords:** angiotensin receptor neprilysin inhibitor, LV dysfunction, meta-analysis, post acute myocardial infarction, ventricular arrhythmia

## Abstract

**Aims:**

Heart failure (HF) is a common and serious complication following acute myocardial infarction (AMI), particularly in patients with impaired left ventricular systolic function [left ventricular ejection fraction (LVEF) < 50%] during hospitalization. Although angiotensin-receptor–neprilysin inhibitor (ARNI) therapy has proven benefit in chronic HF, evidence supporting its initiation during the index AMI admission remains limited. This study systematically evaluated the effects of early in-hospital initiation of ARNI therapy compared with angiotensin-converting enzyme (ACE) inhibitor or angiotensin receptor blocker (ARB) therapy on cardiovascular outcomes in post- AMI patients with impaired systolic function.

**Methods:**

This systematic review and meta-analysis was prospectively registered in the International Prospective Register of Systematic Reviews (PROSPERO CRD420251007504). A comprehensive search was completed on 30 August 2025. We included randomized controlled trials (RCTs) enrolling hospitalized AMI patients with impaired systolic function (LVEF <50%) who initiated ARNI therapy during the index admission. The primary outcome was major adverse cardiovascular events (MACEs), defined as HF hospitalization, all-cause mortality, or recurrent acute coronary syndrome. Secondary outcomes included incidence of ventricular arrhythmia, cardiovascular death, stroke, changes in left-ventricular ejection fraction, NT-proBNP levels, and adverse events (AEs). Prespecified subgroup analyses and meta-regression were performed.

**Results:**

Twelve RCTs comprising 7,539 patients (ARNI: *n* = 3,771; ACEI/ARB: *n* = 3,768) were included, with a mean follow-up of approximately 6 months. Early in-hospital ARNI initiation significantly reduced MACEs [risk ratio (RR) 0.58; 95% CI 0.41–0.83; number-needed-to-treat (NNT) ≈ 7] and ventricular arrhythmia (RR 0.51; 95% CI 0.35–0.75; NNT ≈ 25). ARNI therapy improved LVEF (mean difference +2.54%; 95% CI +1.34 to +3.75) and reduced NT-proBNP levels (mean difference −424 pg/mL; 95% CI −779 to −68). However, ARNI was associated with higher rates of AEs (RR 1.16; 95% CI 1.14–1.19), primarily symptomatic hypotension (RR 1.32; 95% CI 1.20–1.46). Results were consistent across subgroups, with greater incremental benefit observed among patients who underwent successful primary percutaneous coronary intervention.

**Conclusions:**

Among hospitalized post-AMI patients with impaired left ventricular systolic function, early in-hospital initiation of ARNI reduces MACEs, lowers the incidence of ventricular arrhythmia, and improves ventricular function compared with ACEI/ARB therapy, although careful monitoring for hypotension is required. These findings support early in-hospital ARNI adoption in this high-risk population and underscore the need for longer-term outcome studies.

## GRAPHICAL ABSTRACT

**Figure:**
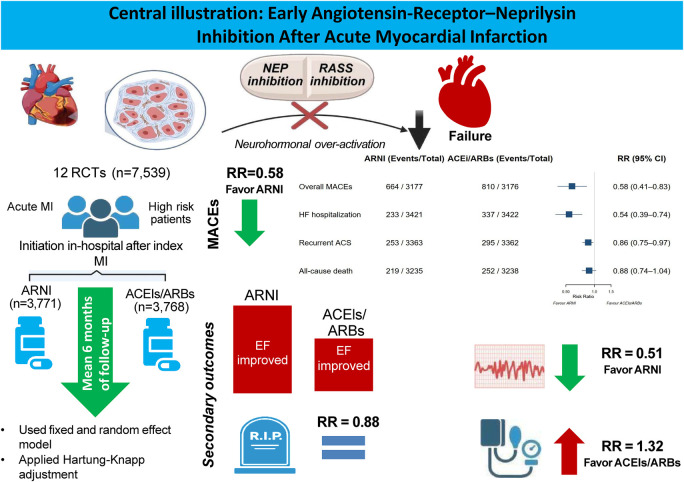

## Introduction

Heart failure (HF) is a frequent and severe complication of acute myocardial infarction (AMI); moreover, a considerable proportion of patients experience new-onset HF within the first year after discharge (1, 2). The risk is up to sixfold higher than the risk of recurrent MI, even in patients with normal left ventricular ejection fraction (LVEF) or without HF during index admission (3)^,^ and its onset remains a major determinant of short- and long-term mortality following AMI (3, 4). The mechanisms driving HF after AMI are complex and heterogeneous, making uniform application of standard HF therapies challenging and sometimes insufficient (5, 6).

Inflammation, including apoptosis and necrosis, plays relevant roles, promoting maladaptive remodeling and HF development following AMI (6–8). The inflammatory processes are also exacerbated by neurohormonal activation, including RAAS, and by insufficient endogenous natriuretic peptide activity, leading to further myocardial injury and maladaptive remodeling (9, 10). This may explain why angiotensin-converting enzyme inhibitors (ACEIs) and angiotensin receptor blockers (ARBs) showed effectiveness in the post-AMI patients (11–13).

Angiotensin receptor–neprilysin inhibitor (ARNI), which includes two components (an angiotensin II receptor blocker and a neprilysin inhibitor), targets these core mechanisms by simultaneously blocking RAAS and augmenting natriuretic peptides, thereby mitigating LV remodeling, improving cardiac function, and delaying HF progression (10). Multiple landmark randomized controlled trials, including PARADIGM-HF and PIONEER-HF, have demonstrated that ARNI therapy confers superior clinical benefits compared with ACEi/ARBs in patients with chronic and acute decompensated heart failure with reduced left ventricular ejection fraction (HFrEF) (14, 15). Nevertheless, patients with new LV dysfunction after AMI were generally excluded from these trials, leaving a substantial knowledge gap regarding the efficacy and safety of ARNI in this population.

The PARADISE-MI trial, the largest investigation to date in the post-AMI setting, demonstrated no significant reduction in the primary composite outcome with ARNI vs. ramipril (16), and current international guidelines consequently provide no specific recommendations for ARNI use in post-AMI patients with LV dysfunction (17). Importantly, this trial exclusively enrolled patients with LVEF <40% and/or pulmonary congestion, leaving a clinically significant population, those with mildly reduced LVEF (40%–49%), without dedicated evidence, despite sharing key pathophysiological features and remaining at considerable cardiovascular risk. This systematic review and meta-analysis was therefore conducted to address this evidence gap by evaluating the efficacy and safety of early in-hospital ARNI initiation in post-AMI patients with LV dysfunction across a broader LVEF spectrum, to inform clinical practice and guide future guideline development.

## Method

Our study was registered in the International Prospective Register for Systematic Reviews (PROSPERO) with the number CRD420251007504.

### Search strategy

We systematically searched six databases: PubMed, Embase, Web of Science, Scopus, CNKI, and Cochrane databases. The most recent search was conducted on August 30, 2025. Specifically, we evaluated the effectiveness of early ARNI administration compared with the current standard of care, which involves the use of ACE inhibitors or ARBs. A combination of keywords and MeSH terms was employed, including ‘acute myocardial infarction’, ‘acute coronary syndrome’, ‘MINOCA’, ‘INOCA’, ‘angiotensin receptor antagonists’, ’Sacubitril’, ‘ARNI’, and ‘Neprilysin inhibitors’ (Detailed search strategy is supplied in Supplementary Table 1).

### Study selection

Studies identified through systematic searches were initially screened by two independent investigators (N.T.Q. and T.T.Q.T.) based on titles and abstracts, following the removal of duplicates. Subsequently, the remaining studies underwent full-text review by the same two investigators to determine eligibility according to the predefined inclusion criteria. Discrepancies between the two reviewers were resolved through consultation with a third investigator (C.Y.H.).

For this review and meta-analysis, studies were eligible if they: (i) were randomized controlled trials (RCTs); (ii) enrolled post-AMI patients have LV dysfunction (ejection fraction < 50%) with or without symptomatic HF; (iii) initiated ARNI early during hospitalization; (iv) compared ARNI with a conventional treatment group; and (v) reported any of the predefined outcomes.

Studies were excluded if they: (i) presented mixed outcome data for ARNI and non-ARNI groups without separate reporting; (ii) were duplicates or pooled analyses with identical outcomes; (iii) were case reports or studies with fewer than 100 participants. Small sample size markedly increases random error, instability of effect estimates, and publication bias, factors well-demonstrated to systematically overestimate treatment effects in cardiovascular meta-analyses (18, 19) and consistent with Cochrane methodology recommendations (20); (iv) were not published in English, Mandarin, or Vietnamese.

**Figure 1:**
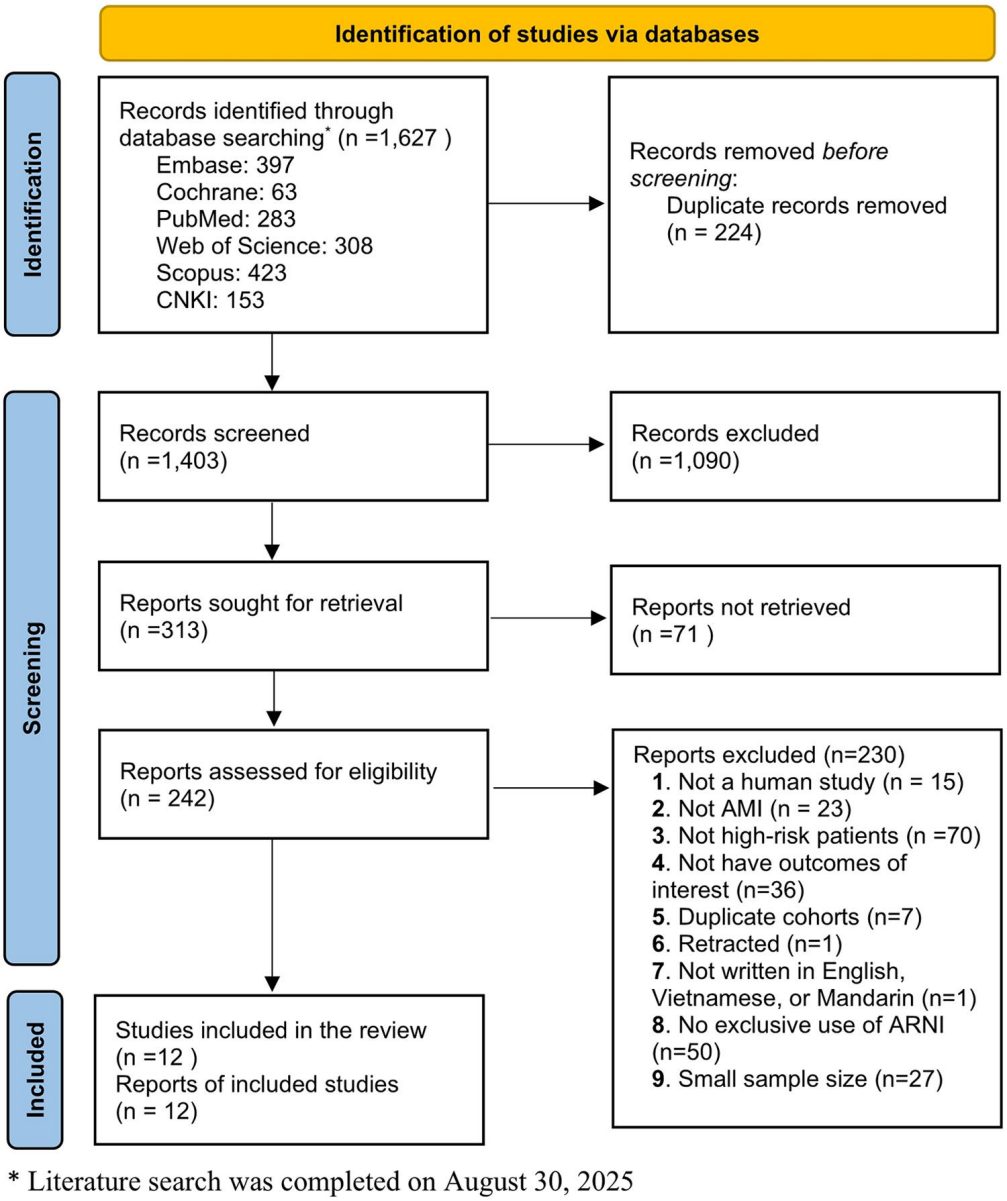
PRISMA flow diagram of study screening and selection. AMI, acute myocardial infarction; ARNI, angiotensin receptor–neprilysin inhibitor.

The PRISMA flowcharts of the study selection process are shown in Figure 1.

### Data extraction

Two authors (Q.N., T.T.Q.T.) separately extracted the data from the included studies, including the author, year, study design, sample size, duration, baseline LVEF, type of acute coronary syndrome, control group, revascularization therapy, and medical treatment (Table 1). Moreover, we collected data regarding baseline characteristics, comorbidities, and HF medication (Supplementary Table 2). Discrepancies among investigators were resolved through consensus, and a third investigator (C.Y.H.) verified the extracted data for accuracy. Furthermore, we emailed the principal investigators for any additional required information.

**Table 1:** Included studies.

No.	Author and year	Total patients	Follow-up (Months)	Centre	Study design	Type of MI	LVEF category	Time of initiation	Revascularization strategy	Control group
1	Lin et al. (38) 2022	109	6	1	RCT	Anterior STEMI	40–49%	In hospital	PCI	Valsartan
2	Abdelnabi et al. (39) 2022	200	6	1	RCT	STEMI	≤40%	In hospital	PCI	Valsartan
3	Rezq et al. (40) 2021	100	6	1	RCT	STEMI	≤40%	38.59 ± 20 h after hemodynamic stability	PCI	Ramipril
4	Yin et al. (41) 2023	142	12	1	RCT	AMI	40–49%	Within 7 days	PCI/thrombolytics	Benazepril
5	Shah et al. (42) 2022	544	8	Multi	RCT	AMI	<50%	Within 4.3 days	PCI/thrombolytics	Ramipril
6	Wang et al. (43) 2021	137	6	1	RCT	Anterior STEMI	40–49%	In hospital	PCI	Enalapril
7	Pfeffer et al. (16) 2021	5,669	22	Multi	RCT	AMI	<50%	Within 4.3 days	PCI/thrombolytics	Ramipril
8	Dong et al. (44) 2022	131	6	1	RCT	Anterior STEMI	40–49%	Within 1 day	PCI	Enalapril
9	Yuan et al. (45) 2024	120	3	1	RCT	AMI	≤40%	In hospital	PCI	Enalapril
10	Fu et al. (46) 2022	126	6	1	RCT	STEMI	≤40%	In hospital	PCI	Fosinopril
11	Zhang et al. (47) 2021	120	6	1	RCT	AMI	≤40%	In hospital	PCI	ACEIs/ARBs
12	Zhu et al. (48) 2024	130	6	1	RCT	STEMI	40–49%	Within 24 h after PCI	PCI	Enalapril

ACEIs, angiotensin-converting enzyme inhibitors; ACS, acute coronary syndrome; AMI, acute myocardial infarction; ARBs, angiotensin receptor blockers; BID, twice daily; HF, heart failure; HFrEF, heart failure with reduced ejection fraction; HFmrEF, heart failure with mildly reduced ejection fraction; MI, myocardial infarction; PCI, percutaneous coronary intervention; RCT, randomized controlled trial; STEMI, ST-elevation myocardial infarction.

### Risk of bias

The Cochrane Risk of Bias ROB 2.0 tool was used to evaluate the quality of randomized trials, categorizing them into three levels: low risk, some concerns, and high risk of bias (21). In cases of disagreement, a consensus approach was used to resolve the issue. Additionally, publication bias was assessed using funnel plots.

### Pre-specified outcomes

The primary outcome was risk of MACEs, defined as a composite of all-cause mortality, HF hospitalization, and recurrent acute coronary syndrome (ACS). Secondary efficacy outcomes included cardiovascular death, ventricular arrhythmia, stroke, intractable angina, and the mean change in LVEF and NT-proBNP after treatment. Adverse events comprised symptomatic hypotension, renal impairment, and hyperkalaemia.

### Statistical analysis

Continuous variables are presented as mean (standard deviation) or median (first and third quartile), while categorical variables are expressed as counts (percentage). Hypothesis testing for superiority was conducted at the two-tailed 0.05 level. Pooled risk ratio (RR) and mean difference (MD) with 95% confidence intervals (CI) were used as summary statistics for outcomes, estimated using a fixed-effects model with generic inverse-variance weighting or a random-effects model if there was high heterogeneity across studies. The I^2^ statistic was used to assess heterogeneity, with low, moderate, and high heterogeneity defined as 0%–25%, 25%–50%, and greater than 50%, respectively. We applied Hartung-Knapp adjustments to improve the robustness of the confidence intervals and manage the heterogeneity across a small number of studies (22). To quantify the treatment effect, Number Needed to Treat (NNT), specified as the Number Needed to Benefit (NNTB) or the Number Needed to Harm (NNTH), was calculated via the absolute risk difference between 2 groups (20). Sensitivity analysis using a leave-one-out approach was conducted to evaluate the impact of individual trials on the effect sizes. Subgroup analyses were performed according to LV dysfunction category, initial medication dose, ethnicity, and control therapy. Additionally, mixed-effects meta-regression analysis with the Hartung-Knapp estimator was applied to assess the impact of predefined variables, including age, gender, baseline LVEF, duration of follow-up, proportion of primary PCI, hypertension, and diabetes, on LVEF change and risk of HF hospitalization. Funnel-plot analysis and Egger's test were conducted to evaluate publication bias and small-study effects. This technique estimates the number of potentially missing studies due to publication bias, imputes these studies, and calculates the overall effect size estimate using both observed and imputed studies (23, 24). Moreover, we assessed the certainty of evidence using the Grading of Recommendations Assessment, Development, and Evaluation (GRADE) framework, implemented via GRADEpro in conjunction with RevMan. This approach incorporated evaluation across five domains—risk of bias, consistency of effect, imprecision, indirectness, and publication bias and facilitated the presentation of results in a Summary of Findings table (25, 26) (Supplementary Table 4).

All statistical analyses and plots were performed using RevMan version 5.2 (The Cochrane Collaboration, The Nordic Cochrane Centre, Copenhagen, Denmark), Risk-of-bias Visualization (Robvis), and R (version 4.4.1, R Foundation for Statistical Computing, Vienna, Austria).

## Results

### Trials and population

Our systematic search retrieved 12 RCTs (including one ECHO substudy) encompassing 7,539 patients. Of the 12 included RCTs, 6 trials reported the prespecified MACE composite and were included in the primary analysis. The remaining 6 trials reported only individual component outcomes or surrogate endpoints and contributed to secondary analyses.

The majority of the included studies were single-center trials from Asia, with a median follow-up of 6 months. Hypertension, diabetes, and dyslipidaemia were the most prevalent comorbidities. Most patients were effectively managed according to guidelines, receiving beta-blockers and undergoing primary PCI on the first day after admission. Nevertheless, mineralocorticoid receptor antagonists and sodium-glucose cotransporter-2 inhibitors were less commonly used. Generally, the initial dose of sacubitril/valsartan was 50 mg twice daily (BID), and was then titrated to 100 mg BID if the patient tolerated it. The risk of bias assessment for all randomized controlled studies is shown in Supplementary Figure 1.

### Primary outcome

**Figure 2:**
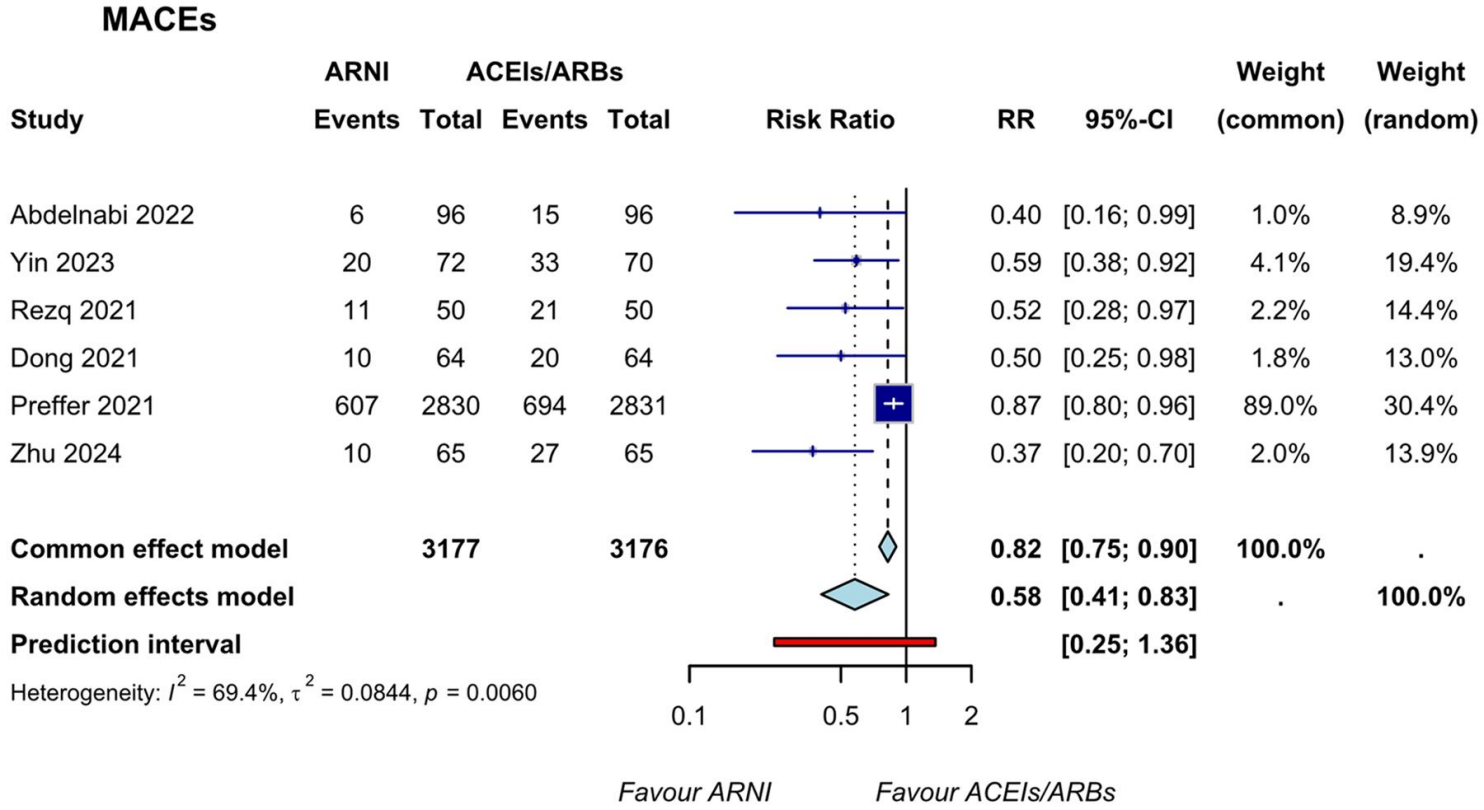
Risk of MACEs. ACEIs, angiotensin–converting enzyme inhibitors; ARBs, angiotensin receptor blockers; ARNI, angiotensin receptor–neprilysin inhibitor; CI, confidence interval; MACEs, major adverse cardiovascular events; RR, risk ratio.

The risk of MACEs pooled from 6 RCTs was significantly lower in the ARNI group than in the conventional group [risk ratio (RR) 0.58; 95% CI 0.41–0.83, NNTB ≈ 7], primarily due to a reduced risk of HF hospitalization (RR 0.54, 95% CI 0.39–0.74, NNTB ≈ 10) (Figure 2 and Supplementary Table 3). GRADE showed a moderate certainty for this finding (Supplementary Table 4). The I^2^ value of 69.4% highlights a high level of heterogeneity. To further understand the factors contributing to this variation, we conducted subgroup analyses focused on baseline LVEF, racial groups, and the initial dose of sacubitril/valsartan. However, all analyses showed no difference between subgroups. (Supplementary Figure 3).

### Secondary efficacy outcomes

**Figure 3:**
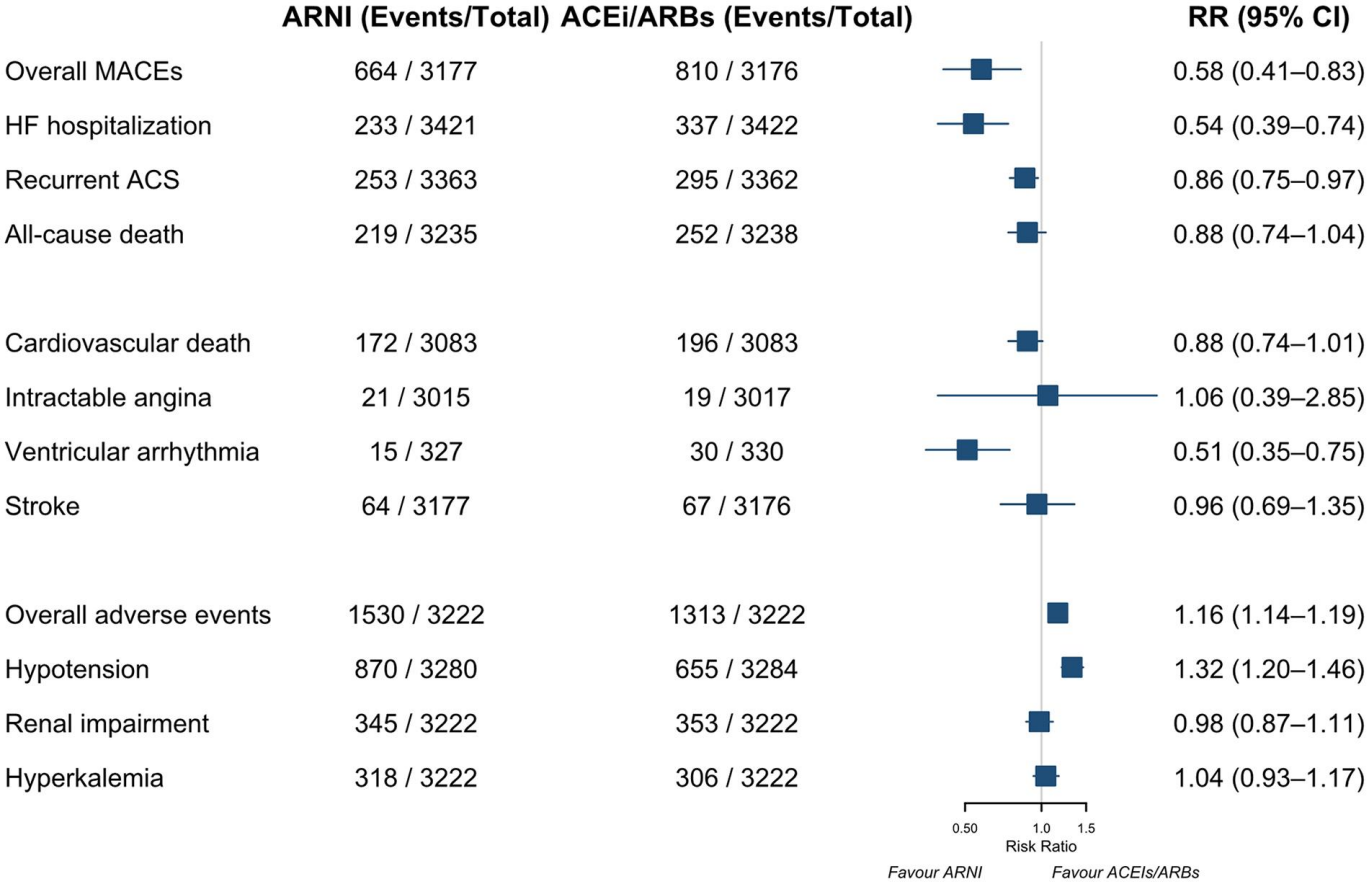
All clinical outcomes and adverse events. ACEIs, angiotensin-converting enzyme inhibitors; ACS, acute coronary syndrome; ARBs, angiotensin receptor blockers; ARNI, angiotensin receptor–neprilysin inhibitor; CI, confidence interval; HF, heart failure; MACEs, major adverse cardiovascular events; RR, risk ratio.

Patients treated with ARNI exhibited a significant reduction of ventricular arrhythmia (RR: 0.51, 95% CI: 0.35–0.75, I^2^ = 0%, NNTB ≈ 25) compared with ACEs/ARBs. However, other clinical endpoints were not statistically different between groups (Figure 3).

The change in LVEF and NT-proBNP level after a minimum of 6 months of follow-up was significantly greater in the ARNI group than in the conventional group (MD: +2.54%; 95% CI 1.34–3.75, I^2^ = 71%) and (MD: −424 pg/mL; 95% CI −779 to – 68, I^2^ = 98.5%), respectively (Supplementary Figures 4, 5). Subgroup analysis based on the type of control therapy suggested that ARNI treatment might exhibit a more pronounced benefit when compared with ARBs than with ACEi (Supplementary Figure 6).

Notably, the results of the meta-regression analysis suggested that the proportion of primary PCI at admission might be associated with EF improvement and lower risk of HF hospitalization in ARNI group compared with conventional therapy. While other study-level factors did not show a statistically significant difference (Supplementary Tables 5, 6).

### Adverse events

The risk of adverse events in the ARNI group was modestly higher than that in the conventional group (RR: 1.16; 95% CI 1.14–1.19, I^2^ = 0%, NNTH ≈ 15), primarily due to the risk of symptomatic hypotension (RR: 1.32; 95% CI 1.20–1.46, I^2^ = 0%, NNTH ≈ 16). There was no difference in renal impairment and hyperkalaemia risk between the two groups (RR: 0.98; 95% CI 0.87–1.11 vs. RR: 1.04; 95% CI 0.93–1.17) (Figure 3).

### Sensitivity analysis

All findings remained consistent after performing a pre-specified sensitivity analysis using the leave-one-out method. Notably, after excluding PARADISE-MI, the largest trial contributing disproportionately to the pooled weight, the risk of MACEs remained robust (RR 0.50; 95% CI 0.39–0.64, I^2^ = 0%, NNT ≈ 6), confirming that this single trial did not materially drive the pooled estimate (Supplementary Figures 7- 20).

### Publication bias

Evaluation of publication bias for all outcomes mostly showed symmetric funnel plots, with no clear evidence of small-study effects, as confirmed by negative Egger's test results (*p* > 0.05). There was a potential risk of publication bias for MACEs, HF hospitalizations, and symptomatic hypotension as assessed visually from the funnel plot and by Egger's test (*p* < 0.05) (Supplementary Figure 2^1^).

We employed the trim-and-fill method to estimate the number of missing studies and adjust the overall effect size accordingly. For the outcomes of MACEs, HF hospitalization, and symptomatic hypotension, we determined that three, five, and four additional studies, respectively, were needed to adjust for potential publication bias (Supplementary Figures 22–S24). Nonetheless, the revised effect size estimates from both observed and imputed studies did not show any significant differences from the original results (Supplementary Tables 7–S9).

## Discussion

This meta-analysis evaluated the largest cohort to date of patients experiencing LV dysfunction after AMI, integrating data from 12 RCTs that compared the efficacy and safety of ARNI with conventional therapy. Our findings indicate that initiating ARNI therapy early is significantly more effective than standard treatment in reducing MACEs, ventricular arrhythmias, improving LVEF, and lowering NT-proBNP concentrations, without elevating the risk of renal impairment or hyperkalemia. Symptomatic hypotension, however, may still occur. Interestingly, exploratory meta-regression analyses suggested a potential interaction between successful primary PCI and the therapeutic effect of ARNI compared with ACEIs/ARBs. However, given the limited number of studies and the inherent risk of ecological bias in study-level analyses, this finding should be considered hypothesis-generating and warrants prospective validation.

These results are consistent with prior meta-analyses by Xiong et al. and Liu et al., which observed that ARNI therapy attenuates LV remodeling, enhances cardiac function, and reduces MACEs in post-AMI patients undergoing primary PCI (27, 28). These benefits likely reflect ARNI's dual mechanism - simultaneous RAAS inhibition and neprilysin-mediated augmentation of natriuretic peptide activity - which has demonstrated consistent improvements in cardiac outcomes across both clinical and experimental settings (29, 30). However, prior meta-analyses have predominantly examined ARNI use in unselected AMI patients following successful primary PCI, leaving important evidence gaps for higher-risk subgroups, particularly those who did not undergo PCI or who presented with symptomatic HF and/or LV dysfunction post-AMI. The PARADISE-MI trial was specifically designed to address this gap. Although the trial confirmed the safety of ARNI and demonstrated a reduction in recurrent HF events, no significant difference in MACE incidence was observed compared with ramipril (HR 0.90; 95% CI 0.78–1.04; *P* = 0.17) (16). Several factors may account for these findings, including suboptimal mineralocorticoid receptor antagonist use, sex-related differences, racial homogeneity of the study population, and a substantial proportion of non-revascularized participants (31, 32).

Our meta-analysis of 12 RCTs included post-AMI patients with LV dysfunction regardless of revascularization status. Nevertheless, the pooled estimate diverges substantially from PARADISE-MI, the largest and most methodologically rigorous trial in the analysis. This discrepancy likely reflects study-level limitations in smaller trials: open-label designs introducing performance bias; surrogate rather than hard clinical endpoints; and predominant single-center Chinese conduct susceptible to publication bias and the small-study effect. Although excluding PARADISE-MI in the sensitivity analysis yielded a consistent estimate (RR 0.50; 95% CI 0.39–0.64; I^2^ = 0%), this must be interpreted cautiously; its exclusion predictably favors a more homogeneous pooled estimate derived solely from methodologically limited smaller trials. Accordingly, the pooled estimate likely represents a best-case scenario that cannot be generalized to all post-AMI patients, and PARADISE-MI should anchor the clinical interpretation of these findings.

Notably, the observed benefits of ARNI therapy were particularly pronounced when combined with successful primary PCI, which may partly account for the more modest outcomes in PARADISE-MI, where approximately 10% of participants did not undergo revascularization (16). Supporting these findings, our prior work demonstrated that myocardial viability influences ARNI response, as patients with non-ischemic cardiomyopathy in whom viable myocardium is relatively preserved achieved significantly greater LVEF improvement compared with those of ischemic etiology, and such improvement was independently associated with favorable clinical outcomes (33). Collectively, these data support the hypothesis that successful revascularization enhances the efficacy of early ARNI therapy by preserving viable myocardium and limiting infarct expansion, thereby creating a favorable substrate for ARNI to exert its anti-remodeling and neurohormonal effects.

Furthermore, our meta-analysis identified a notable reduction in ventricular arrhythmia among patients receiving ARNI treatment compared with those receiving ACEi/ARBs- an observation that has not been highlighted in previous studies. This finding is supported by Diego's study, which has shown that ARNI reduces cardiovascular death and ventricular arrhythmia in HFrEF patients with implantable cardioverter defibrillator (34). In a post-AMI setting, a recent experiment conducted on pigs demonstrated that early administration of ARNI decreased the likelihood of ventricular arrhythmia in the first 30 days by 55% (35). The mechanism might be linked to the role of ARNI in reversing remodeling, systemic inflammatory response modulation, reducing collagen deposition, and improving the electrophysiological properties of the myocardial scar (35).

The timing of ARNI initiation is a critical determinant of therapeutic efficacy, as myocardial fibroblast activation, the primary driver of adverse remodeling, peaks within the first week after AMI and declines progressively over time (36). Accordingly, Docherty et al. found that late ARNI initiation (>3 months post-AMI) failed to significantly reverse remodeling compared with valsartan (37), which underscores the importance of a therapeutic “golden window” during early hospitalization. Our meta-analysis supports early in-hospital ARNI initiation as a strategy to attenuate adverse remodeling, reduce fibrosis, and restore neurohormonal balance in post-AMI management.

A subgroup analysis focusing on control groups, specifically ACEIs and ARBs, revealed that ARNI may be less advantageous when compared with ACEIs than with ARBs, although this difference was not statistically significant. This pattern implies that ACEIs could confer greater benefit than ARBs in post-AMI HF. Ongoing studies and clinical trials are expected to clarify the long-term benefits and optimal deployment of ARNI in this subgroup, thereby informing more nuanced treatment guidelines.

Although ARNI therapy was associated with a significantly increased risk of symptomatic hypotension, drug discontinuation rates did not differ significantly between arms in PARADISE-MI, suggesting hypotension in post-AMI patients was largely manageable without permanent withdrawal. Consistent with current guidelines, ARNI should be initiated at the lowest dose in hemodynamically stabilized patients, with gradual up-titration as tolerated. When symptomatic hypotension occurs, dose reduction and optimization of concomitant vasodilatory agents should be prioritized before considering permanent discontinuation.

This meta-analysis has some limitations. First, ten of 12 included RCTs were single-center studies, predominantly from Chinese institutions, with several unblinded and were published in regional journals. Beyond geographic limitations, meta-epidemiological evidence indicates that unblinded, single-center RCTs systematically overestimate treatment effects. Despite subgroup analyses showing similar LVEF improvements across racial groups, extrapolation to other populations or regions remains tentative. Second, differences in enrolled populations, medication regimens, and study designs contributed to the substantial heterogeneity observed in the primary analysis. Although we performed subgroup analyses, sensitivity analyses, and meta-regression to explore this heterogeneity, residual confounding cannot be excluded and may independently influence findings. Given that the treatment effect observed in PARADISE-MI alone was substantially smaller than the pooled estimate (Supplementary Table 10), the derived NNT of approximately 7 for MACE reduction, while numerically compelling, likely represents an optimistic upper bound rather than an expected effect size in routine clinical practice. Accordingly, this estimate warrants considerable caution and should not be assumed to be generalizable across all post-AMI populations. Third, most of the meta-regressions were analyzed with 10 studies, which might lead to overfitting of the model. The limited diversity in primary PCI proportions across included studies precluded definitive conclusions regarding the synergistic interaction between primary PCI and LVEF improvement; this finding should therefore be considered exploratory and requires prospective validation.

Fourth, the exclusion of trials with fewer than 100 participants, while intended to minimize the influence of unstable small-trial estimates, may limit the comprehensiveness of the evidence base. Accordingly, the reported pooled effect sizes should be interpreted with caution. Additional multicenter studies with large sample sizes across diverse geographical locations are required to improve generalizability.

Finally, the lack of individual-level data and detailed information on clinical characteristics and potential confounding variables, including intervention procedures and specific initiation time of medication, restricted our ability to conduct further analyses. Individual participant data meta-analyses and multinational pragmatic RCTs targeting early ARNI initiation (e.g., the ongoing ASV-AMI trial) (37) are warranted to validate our observations.

## Conclusion

This systematic review and meta-analysis based on the latest data, provides insights into the potential role of early ARNI administration in high-risk patients with AMI-related LV dysfunction. The pooled findings suggest that ARNI may be associated with a reduced risk of MACEs and ventricular arrhythmia, improving cardiac function and NT-proBNP levels, while appearing to preserve renal function. When used in conjunction with primary PCI, early initiation of ARNI may offer incremental benefits over ACEI/ARB therapy for LVEF recovery, though these observations require confirmation in larger, prospective randomized trials before firm conclusions can be drawn. Nonetheless, the increased risk of ARNI-associated symptomatic hypotension requires close monitoring; gradually increasing the dose based on individual tolerance should be considered in clinical practice.

## Data Availability

Publicly available datasets were analyzed in this study. This data can be found here: The original contributions presented in the study are included in the article/Supplementary Material, further inquiries can be directed to the corresponding author.
