# Supplementary material for: Early in-hospital initiation of angiotensin-receptor–neprilysin inhibitor in post-acute myocardial infarction patients with impaired left ventricular systolic function: a systematic review and meta-analysis of randomized controlled trials
Source: Front Cardiovasc Med. 2026 Jul 21;13:1877075. doi: 10.3389/fcvm.2026.1877075 (PMC13433154; doi:10.3389/fcvm.2026.1877075)
Supplement: Supplementary file 1 [file Supplementaryfile1.docx]

[Supplementary Table 1 Database Search Strategy and Results. (Last searching August 30, 2025)	4](#_Toc233396819)

[Supplementary Table 2 Baseline characteristics of participants in the included studies.	6](#_Toc233396820)

[Supplementary Table 3 Numbers needed to treat for all outcomes	9](#_Toc233396821)

[Supplementary Table 4 GRADE of summary finding table	9](#_Toc233396822)

[Supplementary Table 5 Meta-regression about the mean change of LVEF in the long-term (minimum 6 months)	12](#_Toc233396823)

[Supplementary Table 6 Meta-regression about the risk of HF hospitalization	12](#_Toc233396824)

[Supplementary Table 7 Estimated effects of MACEs after applying trim and fill method	33](#_Toc233396825)

[Supplementary Table 8 Estimated effects of HF hospitalization after applying trim and fill method	34](#_Toc233396826)

[Supplementary Table 9 Estimated effects of symptomatic hypotension after applying trim and fill method	35](#_Toc233396827)

[Supplementary Table 10 Comparison of the number needed to treat between PARADISE-MI and the current meta-analysis.	36](#_Toc233396828)

[Supplementary Figure 1 Risk of bias and summary risk of bias from 12 RCTs	11](#_Toc216817335)

[Supplementary Figure 2 Baujat plot illustrates the contribution of each study to the overall result and heterogeneity	12](#_Toc216817336)

[Supplementary Figure 3 The risk of MACEs according to subgroup analyses.	14](#_Toc216817337)

[Supplementary Figure 4 LVEF change after treatment	15](#_Toc216817338)

[Supplementary Figure 5 NT-proBNP level change after treatment	15](#_Toc216817339)

[Supplementary Figure 6 LVEF changes according to subgroup analyses.	18](#_Toc216817340)

[Supplementary Figure 7 Leave-one-out sensitivity analysis of MACEs	18](#_Toc216817341)

[Supplementary Figure 8 Leave-one-out sensitivity analysis of NT-proBNP changing (minimum 6 months)	19](#_Toc216817342)

[Supplementary Figure 9 Leave-one-out sensitivity analysis of LVEF change	19](#_Toc216817343)

[Supplementary Figure 10 Leave-one-out sensitivity analysis of all-cause death	20](#_Toc216817344)

[Supplementary Figure 11 Leave-one-out sensitivity analysis of recurrent ACS	21](#_Toc216817345)

[Supplementary Figure 12 Leave-one-out sensitivity analysis of HF hospitalization	22](#_Toc216817346)

[Supplementary Figure 13 Leave-one-out sensitivity analysis of CV death	23](#_Toc216817347)

[Supplementary Figure 14 Leave-one-out sensitivity analysis of stroke	24](#_Toc216817348)

[Supplementary Figure 15 Leave-one-out sensitivity analysis of ventricular arrhythmia	24](#_Toc216817349)

[Supplementary Figure 16 Leave-one-out sensitivity analysis of intractable angina	24](#_Toc216817350)

[Supplementary Figure 17 Leave-one-out sensitivity analysis of adverse events	25](#_Toc216817351)

[Supplementary Figure 18 Leave-one-out sensitivity analysis of hypotension	25](#_Toc216817352)

[Supplementary Figure 19 Leave-one-out sensitivity analysis of renal impairment	26](#_Toc216817353)

[Supplementary Figure 20 Leave-one-out sensitivity analysis of hyperkalemia	27](#_Toc216817354)

[Supplementary Figure 21 Funnel plots and Egger’s test for all outcomes	35](#_Toc216817355)

[Supplementary Figure 22 Funnel plot of MACEs after using the trim-and-fill method	36](#_Toc216817356)

[Supplementary Figure 23 Funnel plot of HF hospitalization after using the trim-and-fill method	37](#_Toc216817357)

[Supplementary Figure 24: Funnel plot of hypotension after using trim and fill method.	38](#_Toc216817358)

Supplementary Table 1 Database Search Strategy and Results. (Last searching August 30, 2025)

| Database | Search strategy | Search results |
| --- | --- | --- |
| Pubmed | (((sacubitril) or (arni) or (sacubitril plus valsartan) or (neprilysin) or (angiotensin receptor/neprilysin inhibitor))) and ("myocardial infarction" or "acute coronary syndrome" or "st-elevation myocardial infarction" or "non-st elevation myocardial infarction" or "minoca" or "inoca") | 283 |
| Embase | #1: 'sacubitril':ti,ab,kw or 'sacubitril plus valsartan':ti,ab,kw or 'enkephalinase inhibitor':ti,ab,kw or 'arni':ti,ab,kw or 'angiotensin receptor/neprilysin inhibitor':ti,ab,kw  #2: 'myocardial infarction':ti,ab,kw or 'acute coronary syndrome':ti,ab,kw or 'st segment elevation myocardial infarction':ti,ab,kw or 'non st segment elevation myocardial infarction':ti,ab,kw or 'minoca':ti,ab,kw or 'non-obstructive coronary artery disease':ti,ab,kw  #3: #1 and #2 | 397 |
| Web of science | #1: ts=('sacubitril' or 'sacubitril plus valsartan' or 'angiotensin receptor/neprilysin inhibitor' or 'neprilysin' or' arni')  #2: ts=('myocardial infarction' or 'acute coronary syndrome' or 'stemi' or 'nstemi' or 'minoca' or 'inoca' )  #3: #1 and #2 | 308 |
| Cochrane library | #1: "sacubitril plus valsartan" or "arni" or "angiotensin receptor/neprilysin inhibitor" or "neprilysin inhibitor" or "ARNI":ti,ab,kw  #2: "myocardial infarction" or "acute coronary syndrome" or "stemi" or "nstemi" or "minoca" or "inoca":ti,ab,kw | 63 |
| Scopus | #1: title-abs-key ( "sacubitril plus valsartan" or "arni" or "angiotensin receptor/neprilysin inhibitor" or "neprilysin inhibitor" or "ARNI" )  #2: title-abs-key ( "myocardial infarction" or "acute coronary syndrome" or "stemi" or "nstemi" or "minoca" or "inoca" )  #3: #1 and #2 | 423 |
| Cnki | su="sacubitril plus valsartan" or "sacubitril" or "angiotensin receptor/neprilysin inhibitor" or "arni" and su="acute myocardial infarction" or "acute coronary syndrome" or "STEMI" or "NSTEMI" or "minoca" or "inoca" | 153 |

| Supplementary Table 2 Baseline characteristics of participants in the included studies. | | | | | | | | | | | | | | | |
| --- | --- | --- | --- | --- | --- | --- | --- | --- | --- | --- | --- | --- | --- | --- | --- |
|  | Author and year | Baseline EF | SBP (mmHg) | DBP (mmHg) | Initial dose (mg) of SV | Intial dose (mg) of ACEIs/ARBs | PCI (%) | Asian (%) | Age (sd) | Males  (%) | Comorbidities | | Other HF medication (%) | | |
|  |  |  |  |  |  |  |  |  |  |  | Hypertension (%) | Diabetics (%) | Beta-blockers | MRAs | SGLT-2i |
| 1 | Lin  2022 | 45.4 (5) | 130 (19.4) | NA | 24/26mg BID | Valsartan 40mg qd | 100 | 100 | 60.6 (12) | 88 | 41.8 | 27.3 | NA | NA | NA |
| 2 | Abdelnabi 2022 | 34 (4) | NA | NA | 24/26mg BID | Valsartan 40mg BID | 100 | 0 | 58 (11.5) | 66.1 | 69.8 | 49 | NA | NA | NA |
| 3 | Rezq 2021 | 30 (6) | 56.5 (11) | 31.7 (8.7) | 24/26mg BID | Ramipril 5mg qd | 100 | 0 | 54 (11) | 36.5 | 33 | 30 | NA | NA | NA |
| 4 | Yin 2023 | 45.7 (5.4) | 122 (18.1) | 75.1  (12.4) | 24/26mg or 49/51mg BID | Benazepril  2.5 or 5mg qd | 96.4 | 100 | 70 (4) | 59.1 | 58.3 | 44.4 | 88 | NA | 7.7 |
| 5 | Shah  2022 | 42.4 (11.5) | 119 (13) | 79 (10) | 24/26 or 49/51 mg BID | Enalapril 2.5 or 5mg BID | 91 | 4 | 64 (12) | 74 | 68 | 44 | 85 | 48.4 | NA |
| 6 | Wang 2021 | 41.8 (3.1) | 119.2 (12.8) | 75.6 (8.8) | 24/26 or 49/51 mg BID | Enalapril 2.5 or 5mg BID | 100 | 100 | 60 (7) | 77 | 47.1 | 22 | 64.2 | 68.6 | NA |
| 7 | Pfeffer 2021 | 36.5 (9.45) | 121 (13.3) | 73.7 (9.8) | 24/26 or 49/51mg BID | Ramipril 1.25 or 2.5 mg BID | 88 | 16.8 | 64 (12) | 74 | 65.2 | 43.1 | 85.3 | 41.3 | NA |
| 8 | Dong 2022 | 46.4 (9.4) | 110.7 (9.4) | 68.8 (6.4) | 24/26 or 49/51 mg BID | Enalapril 2.5 or 5mg BID | 100 | 100 | 60.3 (9.9) | 79.4 | 44.6 | 23 | 93.9 | 51.2 | NA |
| 9 | Yuan 2024 | 37 (5.8) | NA | NA | 49/51 mg BID | Enalapril  10mg BID | 100 | 100 | 62 (5) | 50 | 25.8 | 22.5 | NA | NA | NA |
| 10 | Fu 2022 | 35.9 (4.35) | NA | NA | 24/26 OR 49/51mg BID | Forsinopril 5 or 1mg qd | 100 | 100 | 65.7 (10.3) | 72.2 | 48.4 | 24.6 | NA | NA | NA |
| 11 | Zhang 2021 | 33.5 (4.2) | NA | NA | 24/26 or 49/51mg BID | ACEIs/ARBs | NA | 100 | 73 (7.7) | 49.2 | 52.5 | 50.8 | NA | NA | NA |
| 12 | Zhu 2024 | 45.8 (4.68) | NA | NA | 49/51mg BID | Enalapril 2.5mg qd | 100 | 100 | 64.7 (6.9) | 63.8 | 39.2 | 23.8 | NA | NA | NA |

*Data are presented as percentage (%), mean (standard deviation), or median (interquartile range)*

*ACEIs, angiotensin-converting enzyme inhibitors; ARBs, angiotensin receptor blockers; BID, two times a day; DBP, diastolic blood pressure; EF, ejection fraction; HF, heart failure; MRAs, mineralocorticoid receptor antagonists; NA, Non-access; PCI, percutaneous coronary intervention; qd, once a day; SBP, systolic blood pressure; SD, standard deviation; SV, sacubitril/valsartan; SGLT-2i, sodium-glucose cotransporter-2 inhibitors*

Supplementary Table 3 Numbers needed to treat for all outcomes

| **Outcome** | **Number of studies** | **Numbers needed to treat (95% CI)** |
| --- | --- | --- |
| MACEs | 6 | NNTB 7 (NNTB 5 to NNTB 18) |
| Recurrent ACS | 9 | NNTB 101 (NNTB 45 to NNTH 391) |
| HF hospitalization | 10 | NNTB 10 (NNTB 7 to NNTB 20) |
| All-cause death | 7 | NNTB 123 (NNTB 61 to NNTH 4401) |
| Stroke | 6 | NNTB 662 (NNTB 118 to NNTH 183) |
| Cardiovascular death | 5 | NNTB 149 (NNTB 68 to NNTH 794) |
| Ventricular arrhythmia | 5 | NNTB 25 (NNTB 14 to NNTB 92) |
| Intractable angina | 4 | NNTH 492 (NNTH 202 to NNTB 1117) |
| Adverse events | 7 | NNTH 15 (NNTH 11 to NNTH 24) |
| Hypotension | 8 | NNTH 16 (NNTH 12 to NTTH 23) |
| Hyperkalemia | 7 | NNTH 574 (NNTH 64 to NNTB 82) |
| Renal impairment | 7 | NNTB 185 (NNTB 51 to NNTH 112) |

*ACS, acute coronary syndrome; MACEs, major adverse cardiovascular events; NNTB, number needed to benefit; NNTH, number needed to harm;*

Supplementary Table 4 GRADE of summary finding table

| Outcome and follow-up | Patients (studies), N | Relative effect (95% CI) | **Absolute effects (95% CI)** | | | Certainty | What happens |
| --- | --- | --- | --- | --- | --- | --- | --- |
|  |  |  | **ACEi/ARBs** | **ARNI** | **Difference** |  |  |
| MACEs | 6353 (6 RCTs) | **RR = 0.58** (0.41 to 0.83) | 255 per 1,000 | **148 per 1,000** (212 to 105) | **107 fewer per 1,000** (from 150 fewer to 43 fewer) | ⨁⨁⨁◯ Moderate^a^ |  |
| Ventricular arrhythmia | 657 (5 RCTs) | **RR = 0.51** (0.35 to 0.75) | 91 per 1,000 | **46 per 1,000** (68 to 32) | **45 fewer per 1,000** (from 59 fewer to 23 fewer) | ⨁⨁⨁⨁ High |  |
| Change of LVEF in long-term | 1591 (10 RCTs) | - | 0 | - | **2.54** (1.34 to 3.75) | ⨁⨁⨁⨁ High | Despite the high heterogeneity observed, meta-regression revealed that the difference in the proportion of primary PCI among studies is the reason. So, the inconsistency is marked as not serious. |
| Change NT-proBNP level between ARNI versus control group after treatment | 882 (7 RCTs) | - | 0 | - | **-416.76** (-554.16 to -279.36) | ⨁⨁◯◯ Low^a,b^ |  |
| All cause of death | 6473 (7 RCTs) | **RR = 0.88** (0.74 to 1.04) | 78 per 1,000 | **68 per 1,000** (81 to 58) | **9 fewer per 1,000** (from 20 fewer to 3 more) | ⨁⨁⨁⨁ High |  |
| Recurrent ACS | 6725 (9 RCTs) | **RR = 0.86** (0.73 to 1.01) | 88 per 1,000 | **75 per 1,000** (89 to 64) | **12 fewer per 1,000** (from 24 fewer to 1 more) | ⨁⨁⨁⨁ High |  |
| HF hospitalization | 6843 (10 RCTs) | **RR = 0.54** (0.39 to 0.74) | 98 per 1,000 | **53 per 1,000** (73 to 38) | **45 fewer per 1,000** (from 60 fewer to 26 fewer) | ⨁⨁⨁◯ Moderate^a,c^ |  |
| Stroke | 6353 (6 RCTs) | **RR = 0.96** (0.69 to 1.36) | 21 per 1,000 | **20 per 1,000** (29 to 15) | **1 fewer per 1,000** (from 7 fewer to 8 more) | ⨁⨁⨁⨁ High |  |
| Adverse events | 6444 (7 RCTs) | **RR = 1.16** (1.14 to 1.19) | 408 per 1,000 | **473 per 1,000** (485 to 465) | **65 more per 1,000** (from 57 more to 77 more) | ⨁⨁⨁⨁ High |  |
| Renal impairment | 6444 (7 RCTs) | **RR = 0.98** (0.85 to 1.13) | 107 per 1,000 | **105 per 1,000** (121 to 91) | **2 fewer per 1,000** (from 16 fewer to 14 more) | ⨁⨁⨁◯ Moderate^c^ |  |
| Hypotension | 6564 (8 RCTs) | **RR = 1.32** (1.21 to 1.44) | 199 per 1,000 | **263 per 1,000** (287 to 241) | **64 more per 1,000** (from 42 more to 88 more) | ⨁⨁⨁◯ Moderate^c^ |  |
| Rate of hyperkalemia | 6444 (7 RCTs) | **RR = 1.04** (0.90 to 1.21) | 95 per 1,000 | **99 per 1,000** (115 to 85) | **4 more per 1,000** (from 9 fewer to 20 more) | ⨁⨁⨁⨁ High |  |
| CV death | 6166 (5 RCTs) | **RR = 0.88** (0.72 to 1.07) | 64 per 1,000 | **56 per 1,000** (68 to 46) | **8 fewer per 1,000** (from 18 fewer to 4 more) | ⨁⨁⨁⨁ High |  |

a. A serious inconsistency rating was given due to high heterogeneity, as shown by the I² and Cochran Q p-value.

b. A low rating for precision was assigned due to the broad confidence interval observed.

c. A publication bias is detected via funnel plot and Egger's test (p < 0.05)

*ACEIs, angiotensin-converting enzyme inhibitors; ARBs, angiotensin receptor blockers; ARNI, angiotensin receptor–neprilysin inhibitor; CI, confidence interval; MD, mean difference; HF, heart failure; LVEF, left ventricular ejection fraction; RR, risk ratio; RCTs, randomized controlled trials;*

***Supplementary Table 5 Meta-regression about the mean change of LVEF in the long-term (minimum 6 months)***

| Moderators | Number of studies | Β coefficient (95% Confidence Interval) | P-value |
| --- | --- | --- | --- |
| Age | 10 | 0.07 (-0.16 to 0.29) | 0.51 |
| Male | 10 | -0.04 (-0.12 to 0.04) | 0.09 |
| Baseline LVEF | 10 | - 0.08 (-0.28 to 0.14) | 0.42 |
| Proportion of PCI | 10 | 0.40 (0.15 to 0.66) | ***0.007*** |
| Follow-up duration | 14 | -0.03 (-0.43 to 0.37) | 0.87 |
| Hypertension proportion | 10 | -0.03 (-0.14 to 0.07) | 0.49 |
| Diabetes proportion | 10 | 0.02 (-0.1 to 0.14 ) | 0.66 |

Supplementary Table 6 Meta-regression about the risk of HF hospitalization

| Moderators | Number of studies | Β coefficient (95% Confidence Interval) | P-value |
| --- | --- | --- | --- |
| Age | 10 | -0.005 (-0.07 to 0.06) | 0.86 |
| Male | 10 | 0.007 (-0.016 to 0.03) | 0.48 |
| Baseline LVEF | 10 | - 0.01 (-0.07 to 0.05) | 0.68 |
| Proportion of PCI | 10 | - 0.05 (-0.08 to -0.02) | ***0.004*** |
| Hypertension proportion | 10 | 0.02 (-0.01 to 0.04) | 0.18 |
| Diabetes proportion | 10 | 0.009 (-0.03 to 0.05) | 0.62 |

*ACEIs, angiotensin-converting enzyme inhibitors; ARBs, angiotensin receptor blockers; ARNI, angiotensin receptor/neprilysin inhibitor; CI, confidence interval; HF, heart failure; LVEF, left ventricular ejection fraction;*

Supplementary Figure 1 Risk of bias and summary risk of bias from 12 RCTs

Supplementary Figure 2 Baujat plot illustrates the contribution of each study to the overall result and heterogeneity

(A)

(B)

(C)

Supplementary Figure 3 The risk of MACEs according to subgroup analyses.

(A) Subgroup based on baseline LVEF.
(B) Subgroup based on ethnicity.
(C) Subgroup based on the initiation dose of ARNI.

*ACEIs, angiotensin-converting enzyme inhibitors; ARBs, angiotensin receptor blockers; ARNI, angiotensin receptor–neprilysin inhibitor; CI, confidence interval; HF, heart failure; LVEF, left ventricular ejection fraction; RR, risk ratio.*

Supplementary Figure 4 LVEF change after treatment

*ACEIs, angiotensin-converting enzyme inhibitors; ARBs, angiotensin receptor blockers; ARNI, angiotensin receptor–neprilysin inhibitor; CI, confidence interval; HF, heart failure; LVEF, left ventricular ejection fraction; RR, risk ratio.*

Supplementary Figure 5 NT-proBNP level change after treatment

*ACEIs, angiotensin-converting enzyme inhibitors; ARBs, angiotensin receptor blockers; ARNI, angiotensin receptor–neprilysin inhibitor; CI, confidence interval; HF, heart failure; LVEF, left ventricular ejection fraction; RR, risk ratio.*

(A)

(B)

(C)

(D)

Supplementary Figure 6 LVEF changes according to subgroup analyses.

(A) Subgroup based on types of comparison
(B) Subgroup based on LVEF
(C) Subgroup based on initiation dose of ARNI
(D) Subgroup based on comparison therapy with ACEi or ARBs

*ACEIs, angiotensin-converting enzyme inhibitors; ARBs, angiotensin receptor blockers; ARNI, angiotensin receptor–neprilysin inhibitor; CI, confidence interval; HF, heart failure; LVEF, left ventricular ejection fraction; RR, risk ratio.*

Supplementary Figure 7 Leave-one-out sensitivity analysis of MACEs

Supplementary Figure 8 Leave-one-out sensitivity analysis of NT-proBNP changing (minimum 6 months)

Supplementary Figure 9 Leave-one-out sensitivity analysis of LVEF change

Supplementary Figure 10 Leave-one-out sensitivity analysis of all-cause death

Supplementary Figure 11 Leave-one-out sensitivity analysis of recurrent ACS

Supplementary Figure 12 Leave-one-out sensitivity analysis of HF hospitalization

Supplementary Figure 13 Leave-one-out sensitivity analysis of CV death

Supplementary Figure 14 Leave-one-out sensitivity analysis of stroke

Supplementary Figure 15 Leave-one-out sensitivity analysis of ventricular arrhythmia

Supplementary Figure 16 Leave-one-out sensitivity analysis of intractable angina

Supplementary Figure 17 Leave-one-out sensitivity analysis of adverse events

Supplementary Figure 18 Leave-one-out sensitivity analysis of hypotension

Supplementary Figure 19 Leave-one-out sensitivity analysis of renal impairment

Supplementary Figure 20 Leave-one-out sensitivity analysis of hyperkalemia

*ACEIs, angiotensin-converting enzyme inhibitors; ARBs, angiotensin receptor blockers; ARNI, angiotensin receptor–neprilysin inhibitor; CI, confidence interval; CV, cardiovascular; HF, heart failure; LVEF, left ventricular ejection fraction; RR, risk ratio.*

Supplementary Figure 21 Funnel plots and Egger’s test for all outcomes

*ACEIs, angiotensin-converting enzyme inhibitors; ARBs, angiotensin receptor blockers; ARNI, angiotensin receptor/neprilysin inhibitor; ACS, acute coronary syndrome; MACEs; major adverse cardiovascular events; MD, mean difference; RR, risk ratio; SE, standard error;*

Supplementary Figure 22 Funnel plot of MACEs after using the trim-and-fill method

Supplementary Table 7 Estimated effects of MACEs after applying trim and fill method

| Number of added studies | Estimate (RR) | Standard error (SE) | p-value | 95% CI-Lower | 95%CI-upper |
| --- | --- | --- | --- | --- | --- |
| 3 | 0.8 | 0.08 | <.001 | 0.65 | 0.96 |

Three studies are missing on the right side, which were imputed to adjust for potential publication bias. After applying the trim-and-fill method, the estimated effect remains significant (RR=0.8, p < .001), meaning ARNI treatment is still superior to conventional therapy in reducing the risk of MACEs.

Supplementary Figure 23 Funnel plot of HF hospitalization after using the trim-and-fill method

Supplementary Table 8 Estimated effects of HF hospitalization after applying trim and fill method

| Number of added studies | Estimate (log Risk Ratio) | Standard error (SE) | p-value | 95% CI-Lower | 95%CI-upper |
| --- | --- | --- | --- | --- | --- |
| 5 | 0.79 | 0.08 | <.001 | 0.64 | 0.94 |

Five studies are missing on the right side, which were imputed to adjust for potential publication bias. After applying the trim-and-fill method, the estimated effect remains significant (RR=0.79, p <.001), meaning ARNI treatment is still superior to conventional therapy in reducing the risk of HFH.

Supplementary Figure 24 Funnel plot of hypotension after using trim and fill method.

Supplementary Table 9 Estimated effects of symptomatic hypotension after applying trim and fill method

| Number of added studies | Estimate (log Risk Ratio) | Standard error (SE) | p-value | 95% CI-Lower | 95%CI-upper |
| --- | --- | --- | --- | --- | --- |
| 4 | 1.37 | 0.24 | <.001 | 0.9 | 1.85 |

Four studies are missing on the left side, which were imputed to adjust for potential publication bias. After applying the trim-and-fill method, the estimated effect remains significant (RR= 1.37, p < .001), meaning the risk of symptomatic hypotension in the ARNI group is higher than in the control group.

Supplementary Table 10 Comparison of the number needed to treat between PARADISE-MI and our meta-analysis.

| **Study** | **RR** | **95% CI** | **Number needed to treat (range)** |
| --- | --- | --- | --- |
| PARADISE-MI alone | 0.9 | 0.78-1.04 | 82 |
| Our pooled meta-analysis | 0.58 | 0.41-0.83 | 7 |
